# Online Stress Measurement During Laser-aided Metallic Additive Manufacturing

**DOI:** 10.1038/s41598-019-39849-0

**Published:** 2019-05-21

**Authors:** Yi Lu, Guifang Sun, Xianfeng Xiao, Jyoti Mazumder

**Affiliations:** 1 College of Mechanical Engineering, Southeast University Nan Jing, Nan Jing, 210000 China; 2 Department of Mechanical Engineering, College of Engineering, University of Michigan, Ann Arbor, 48109-2136 USA; 3 College of Mechanical and Vehicle Engineering, Hunan University, Changsha, 410082 China

**Keywords:** Mechanical engineering, Software

## Abstract

*In situ* measurement of residual stress is a challenge, and it is a source of many defects during additive manufacturing (AM). Usually, postmortem measurement is too late to save the product once a defect appears. Most of the existing technologies are predictive simulations and postmortem analysis. However, these technologies cannot directly reflect the stress evolution during the fabrication process. This paper introduces a computer vision-based stress monitoring system combined with finite element method (FEM) technology to estimate the stress development inside of the deposition layer. The system uses a CCD camera and a line laser beam to measure the height of the melt pool and solidified layer, forms a real-time FEM model, and uses the surface displacement between the two states to calculate the stress development during the solidification process. The results show that there is no obvious shape change after solidification. The shape of the melt pool and its solid state is similar. The stress distribution obtained through online monitoring is similar to that from the traditional thermal-stress simulation.

## Introduction

Laser-aided metallic additive manufacturing uses a laser as a heat source to melt the substrate, deliver metal powder into the melt pool coaxially, and deposit it on the substrate to form various shapes required by the user. This method has broad applications for the repairing, cladding and manufacturing^1^ of engineering components and artistic pieces.

However, due to the complicated thermal history of laser-based AM processes, uneven thermal contraction and expansion occur, which generate a massive amount of residual stress inside the solidified material^2^. These stresses may exceed the strength of the material and can cause undesired distortion and cracking^3^. The stress-caused defects often occur slowly and gradually without obvious symptoms and may break out with minimal external stimulus. Therefore, controlling the stress during the AM process is essential.

Due to the high temperature, plasma and sparks are generated during the laser-based AM process. It is difficult to position a traditional apparatus to measure the stress during manufacturing. Currently, the most common methods are simulation and postmortem stress examination. The numerical simulation begins with pure temperature calculation, through 2D to 3D models^4–7^. Then, based on the temperature history, the stress calculation developed by P. Zhang^8^ and P. Farahmand^9^ is used to calculate the stress field of the laser cladding layer based on the temperature field. For a more accurate calculation, H. Qi^10^ and Xinran Zhao^11^ added solidification phase changes to the model to make the result more closely resemble the experimental data. Another method to measure residual stress is to do postmortem analysis after manufacturing and analyse it with the laser parameters. R. J. Moat^12^ and Pratt, P^13^ use neutron diffraction to measure the stress in direct metal deposition (DMD) manufactured blocks. U. de Oliveira^14^ uses X-ray diffraction to measure the residual stress in Co-based laser clad layers. Although numerical prediction and post-experimental measurement can help us to better understand the DMD process, they can hardly save the product when defects lead to failure. In addition, there is limited room to integrate X-ray or neutron diffraction equipment into the DMD system and insufficient time to do the online simulation based on the traditional method. Another way to monitor the process is to evaluate the strain evolution on the substrate. Andreas^15^ uses a LVDT (linear variable differential transformer) gauge to measure the out-of-plane deformation at the centre of the bottom surface of the substrate. However, this method cannot directly reflect the real condition inside the deposition layer. M. Biegler^16^ applied digital image correlation (DIC) technology to measure the distortion on the substrate. This method must spray a cover layer on the target area, which makes it easier to detect the distortion. However, the cover layer can hardly be applied to the deposition layer because its shape is continuously changing and a relatively high temperature makes it very hard to form a stable optical coverage layer.

In this article, we develop a new method to monitor the stress evolution during the DMD process. Based on computer vision and FEM analysis, this method can make a quick prediction of the stress generated during the laser deposition process.

## Methods

**Figure 1:**
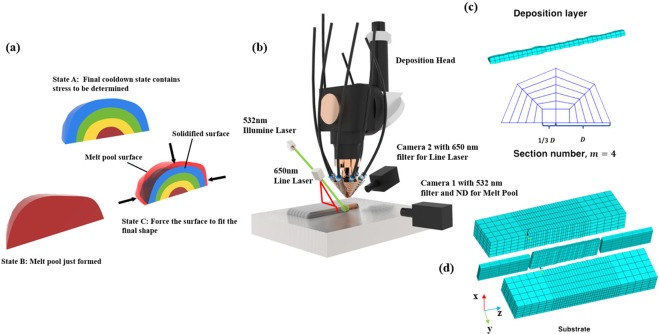
Schematic of real-time stress measurement. (**a**) The principle of displacement method, (**b**) real-time measuring system, (**c**) deposition layer model based on molten pool shape, (**d**) substrate model.

The method we call it displacement method. As illustrated in Fig. 1, state A represents the final solidified material, it is a state which contains the residual stress that we want to determine. B is the shape of the molten pool when it just forms. At this time, the material is in a liquid state; although it contains certain forces and stresses in it, such properties are negligible compared to those of the solid state. We can assume that, in this state, the stress has not formed yet and can take this state as a stress-free state. In the actual manufacturing process, the shape of the state B is contracting to state A due to the cooling of the melt pool. This process can be simulated by forcing the surface of state B to fit state A. The stress generated in this process is saved in state C. Due to the stress superposition principle, the stress in state A can be expressed as:

1 $${\rm{\sigma }}({\rm{A}})(x,y,z)={\rm{\sigma }}({\rm{B}})(x,y,z)+{\rm{\sigma }}({\rm{C}})(x,y,z)$$

According to the hypothesis, σ (B) is 0 and σ (A) ≈ σ (C).

Based on our hypotheses, the stress development can be calculated through the difference between the surface of the melt pool and the solidified material. To obtain these data, we use computer vision to help us take the measurements.

The online measurement layout is shown in Fig. 1b. The melt pool height is recorded by a camera that is set perpendicular to the manufacturing laser. To visualize the melt pool, which is shrouded by bright plasma plume, a 532 nm filter and a neutral density filter (ND) are mounted in front of the camera lens. A 532 nm laser (8 W) is used to illuminate the melt pool area.

The height of the solidified layer is measured by a line laser (650 nm) point 3 mm behind the melt pool. Another camera (camera 2) is used to record the shape change of the line laser. To filter out the light of plasma and 532 nm laser, a 650 nm filter is mounted in front of camera 2. Both cameras are Micro-Mac ® 8SG, 60 fps, and the shutter in camera 1 was set to 1/500 seconds.

The online stress measurement is based on the following steps:

1. Record the height of the melt pool and the deposition layer using camera 1 and camera 2 and send the information to the computer to process the data.
2. Build the cross-section of the melt pool and the deposition layer. In this article, we assume the cross-section of the deposition layer is a semicircle and build the section according to the height of the melt pool and deposition layer.
3. Mesh the cross section of the melt pool and the deposition layer and build the FEM model of the melt pool. Build the substrate element and nodes according to the shape of the melt pool.
4. Calculate the surface displacement between the melt pool and solidified layer. Add displacement on the surface nodes.
5. Add a new melt pool and repeat steps 1–4. After each step, save the result of the last step, substitute the result of the last step, and add displacement on the new deposited part of the materials. Continue the calculation until the process is over.

Before doing the measurement, the camera should be calibrated. When mounted with the lens, the image from the camera may be distorted. Due to the curvature of the lens, light rays near the edges of the lens tend to bend more than those passing through the centre of the lens. The distortion caused by the lens can be described as:

2 $$\{{k}_{1}{k}_{2}{k}_{3}{p}_{1}{p}_{2}\}$$

where *k*_1_, *k*_2_, *k*_3_ are radial distortion coefficients of the lens, and *p*_1_, *p*_2_ are tangential distortion coefficients of the lens. With these parameters, the distortion can be corrected by the equation^17^:

3 $${x}_{r}=(1+{k}_{1}{r}^{2}+{k}_{2}{r}^{4}+{k}_{3}{r}^{6}){x}_{i}+2{p}_{1}{x}_{i}{y}_{i}+{p}_{2}({r}^{2}+2{x}_{i}^{2})$$

4 $${y}_{r}=(1+{k}_{1}{{\rm{r}}}^{2}+{k}_{2}{r}^{4}+{k}_{3}{r}^{6}){x}_{i}+{p}_{1}({{\rm{r}}}^{2}+2{{\rm{y}}}_{i}^{2})+2{p}_{2}{x}_{i}{y}_{i}$$

where *x*_r_ and *y*_r_ are the distorted coordinate the images, *x*_i_ and *y*_i_ are undistorted pixel locations, and *r* is the radial distance of an undistorted pixel point to the centre of the image:

5 $${r}^{2}={x}_{i}^{2}+{y}_{i}^{2}$$

In this article, we use a chequerboard pattern and MATLAB “Single Camera Calibrator” toolbox to perform the calibration.

**Figure 2:**
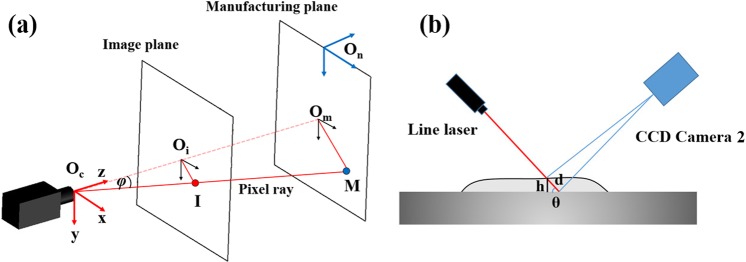
The measurement of melt pool height and solidified layer. (**a**) The relationship between pixels in the image plane and the coordinates in the real world, (**b**) Calculating the height through the line laser.

To measure the height in the camera image, the relationship between pixels coordinates on the filmed image and the real-world coordinates should be clear. As shown in Fig. 2a, based on the position of camera and nozzle, the camera coordinates and the nozzle coordinates are built, respectively. *O*_*c*_ is the origin of the camera coordinates, and *O*_*n*_ is the origin of the nozzle coordinates. *O*_*i*_ is the centre of the image plane, and *O*_*m*_ is the intersection point of the ray $\mathop{{O}_{c}{O}_{I}}\limits^{\rightharpoonup }$ and the manufacturing plane. In camera coordinates, the length of *O*_*m*_*M* can be calculated as:

6 $${O}_{m}M=\frac{{O}_{i}I}{{O}_{c}I}{O}_{c}M$$

7 $${O}_{m}M=\frac{{O}_{c}{O}_{m}}{cos\theta }$$

where *φ* is the angle between *O*_*c*_*O*_*i*_ and *O*_*c*_*I*. The coordinate of M in camera coordinates is M (*x*_c_, *y*_c_, *z*_c_), and then, the coordinate of M in nozzle coordinates (*x*_n_, *y*_n_, *z*_n_) can be written as:

8 $$(\begin{array}{c}{x}_{n}\\ {y}_{n}\\ {z}_{n}\end{array})=(\begin{array}{c}{x}_{c}\\ {y}_{c}\\ {z}_{n}\end{array})+{T}_{CN}$$

where *T*_CN_ is the transaction matrix from camera coordinates to nozzle coordinates.

As shown in Fig. 2b, the line laser is set above the deposited layer. CCD Camera 2 is set behind the line laser. The incident angle of line laser is *θ*, based on the calibration in the previous section, the height of h in Fig. 2b can be measured. Then, the real height of the deposited layer can be calculated as:

9 $$h=d\cdot sin\theta $$

We select 30 frames from the recorded video. According to the number of frames, the deposited layer is divided into the same number of sections as the frames. As shown in Fig. 1c, the section of deposition layer has first meshed. Then, the nodes in each section connect to form elements. The substrate is divided into 5 parts, and the mesh in each part has been optimized to accommodate the random edge of the deposited layer (Fig. 1d). To save computation time, in the displacement method, we take 9 elements in each section of the deposition layer and five layers in the substrate so that the total number of elements is 17738.

An offline thermal-stress simulation is made for comparison in this article. The thermal-stress analysis has two steps. First is a non-linear transient thermal analysis, which calculates the temperature field during laser deposition. Second is a transient stress analysis, based on the temperature field obtained from the previous step. The geometry model of 3D FEA thermal-stress analysis is also based on the measured shape of the deposition layer.

For thermal modelling, the thermal equilibrium equation satisfies the classical 3D heat conduction equation given by^9^

10 $$\rho c\frac{\partial T}{\partial t}=\frac{\partial }{\partial x}(k\frac{\partial T}{\partial x})+\frac{\partial }{\partial y}(k\frac{\partial T}{\partial y})+\frac{\partial }{\partial z}(k\frac{\partial T}{\partial z})+Q$$

where *ρ* is the material density (kg/m^3^), c is the specific heat capacity (J/kg K), *T* is the temperature, t is the interaction time, *k* is thermal conductivity (W/mK), and *Q* = (*x*, *y*, *z*, *t*) is the volumetric heat generation (W/m^3^).

The latent heat of fusion is simulated by an artificial increase in the liquid specific^18^ heat and the relationship between the enthalpy, H, density, p, and specific heat, c, and can be written as

11 $$H={\int }^{}\rho c(T)dT$$

The laser heat source used in this model is written as

12 $${\rm{Q}}(r)=\frac{2AP}{\pi {\omega }^{2}}\exp (-\frac{2{r}^{2}}{{\omega }^{2}})$$

where r is the radial distance from the beam centre, *P* is the laser power, and *ω* is the radius of the beam. *A* is the absorptivity of the powder material, which in the present work is *A* = 0.75^19^.

For isotropic material, the stress-strain relationship can be written in Cartesian coordinates as follows^20^:

13 $$\begin{array}{c}{\varepsilon }_{xx}=\frac{1}{E}[{\sigma }_{xx}\times v({\sigma }_{yy}+{\sigma }_{zz})]+{\alpha }_{e}{\rm{\Delta }}T\\ {\varepsilon }_{yy}=\frac{1}{E}[{\sigma }_{yy}\times v({\sigma }_{xx}+{\sigma }_{zz})]+{\alpha }_{e}{\rm{\Delta }}T\\ {\varepsilon }_{zz}=\frac{1}{E}[{\sigma }_{zz}\times v({\sigma }_{xx}+{\sigma }_{yy})]+{\alpha }_{e}{\rm{\Delta }}T\end{array}\,$$

14 $${{\epsilon }}_{xy}=\frac{1+v}{E}{\sigma }_{xy}\,\,\,{{\epsilon }}_{xz}=\frac{1+v}{E}{\sigma }_{xz}\,\,\,{{\epsilon }}_{yz}=\frac{1+v}{E}{\sigma }_{yz}$$

where E, v and *a*_*e*_ are the modulus of elasticity, Poisson’s ratio and coefficient of thermal expansion, respectively. Δ*T* represents a temperature rise at a point (x, y, z) at time t with respect to that at t = 0, corresponding to a stress-free condition.

The effective stress can be given as,

15 $${\sigma }_{eff}=\sqrt{{\sigma }_{1}^{2}+{\sigma }_{2}^{2}+{\sigma }_{3}^{2}}+2v({\sigma }_{2}{\sigma }_{2}+{\sigma }_{1}{\sigma }_{3}+{\sigma }_{2}{\sigma }_{2})$$

where *σ*_1_, *σ*_2_, and *σ*_3_ are the three principal stresses. The VonMises equivalent stress can be written as:

16 $${\sigma }_{eqv}=\sqrt{\frac{1}{2}[{({\sigma }_{2}\times {\sigma }_{2})}^{2}+{({\sigma }_{2}\times {\sigma }_{3})}^{2}+{({\sigma }_{3}\times {\sigma }_{1})}^{2}]}$$

## Results and Discussion

**Figure 3:**
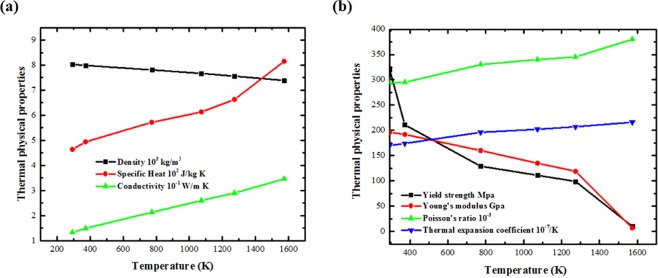
Temperature-dependent (**a**) thermal-physical properties and (**b**) thermal-mechanical properties of AISI 316 stainless steel.

**Figure 4:**
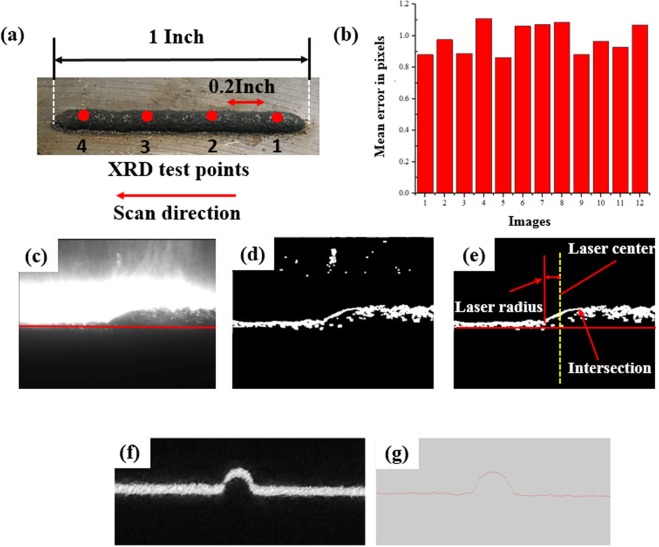
The melt pool image, (**a**) deposited sample, (**b**) mean error in pixels, (**c**) original image, (**d**) edge detection, (**e**) apply threshold.

The powder used in this article is 1236 F/FE-271(Praxair USA), and its composition is close to that of AISI 316 L stainless steel. The thermal properties can be found in article^21^ and are given in Fig. 3. The shielding gas was nitrogen, with a flowing velocity of 20 CFH. To make the measurement easier, we used a low scan speed of 0.5 inch/s and a 1500 W laser to create a relatively large deposition layer. The sample is shown in Fig. 4a. For camera calibration, we take 12 different camera positions to estimate the extrinsic parameters. Fig. 4b shows the re-projection errors of the camera calibration. The error varies in different conditions but in a relatively small range. The average mean error is 0.981 ± 0.112 pixels or 0.018 ± 0.002 mm. The final residual stress of the sample is measured through XRD method (Proto LXRD residual stress measuring instrument, Proto Canada). The Cr target was used in the experiment.

As shown in Fig. 4c–e, the melt pool height measurement is based on the following steps: first, convert the image into the greyscale, and then, use the Prewitt edge detection method to distinguish edges. Since we know the manufacturing laser position and the laser radius, the concerned area is restricted in the laser-irradiated area. Once the molten pool is identified, measure the 10 highest pixels in the selected area and take the average height of them as the height of the molten pool.

For line laser, Fig. 4 f is the camera image of line laser illuminating on the deposited layer. The real location of the deposited layer contour is located in the centre of the line laser, i.e., the middle point on the vertical direction along each column of image pixels. The measured edge is shown in Fig. 4g.

**Figure 5:**
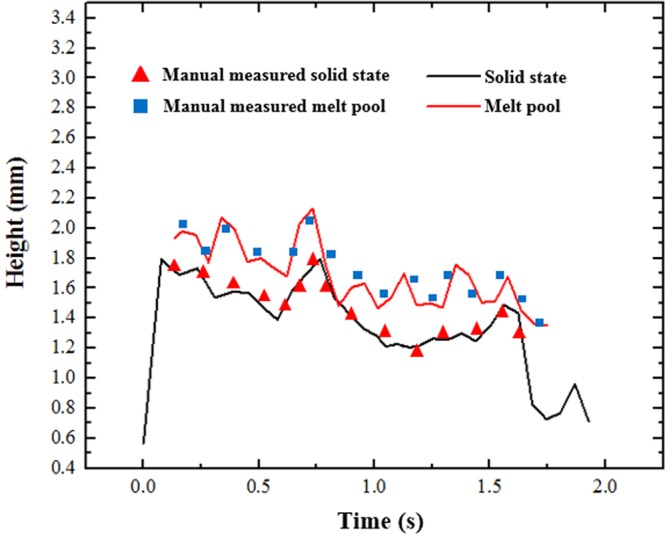
The height of melt pool and solidified layer.

Figure 5 shows the height of the melt pool and the deposition layer. To validate the measurement accuracy, we also measure the height manually through the images captured during the process. The average error in the melt pool and solid state are 0.048 ± 0.032 mm and 0.034 ± 0.031 mm, respectively, or 2.43 ± 1.62 and 1.49 ± 1.32 pixels. The results show that the shape of deposition layer does not change dramatically from the melt pool to the solid state and that the surface curvature of the two states is similar. The height of the solidification layer is slightly lower than that of the melt pool. We also use the line laser to measure the height change during the cooling process, i.e., set the line laser 3 mm behind the melt pool, 10 mm behind the melt pool, and then re-scan the shape after the deposition process. Furthermore, we also measured the height change in the recorded video frame by frame. The results showed that, after molten pool pass, the height of the solidification material did not change significantly, that is to say, the deformation happened less than 0.0166 s (the frame rate is 60fps). This result is consistent with our hypothesis.

**Figure 6:**
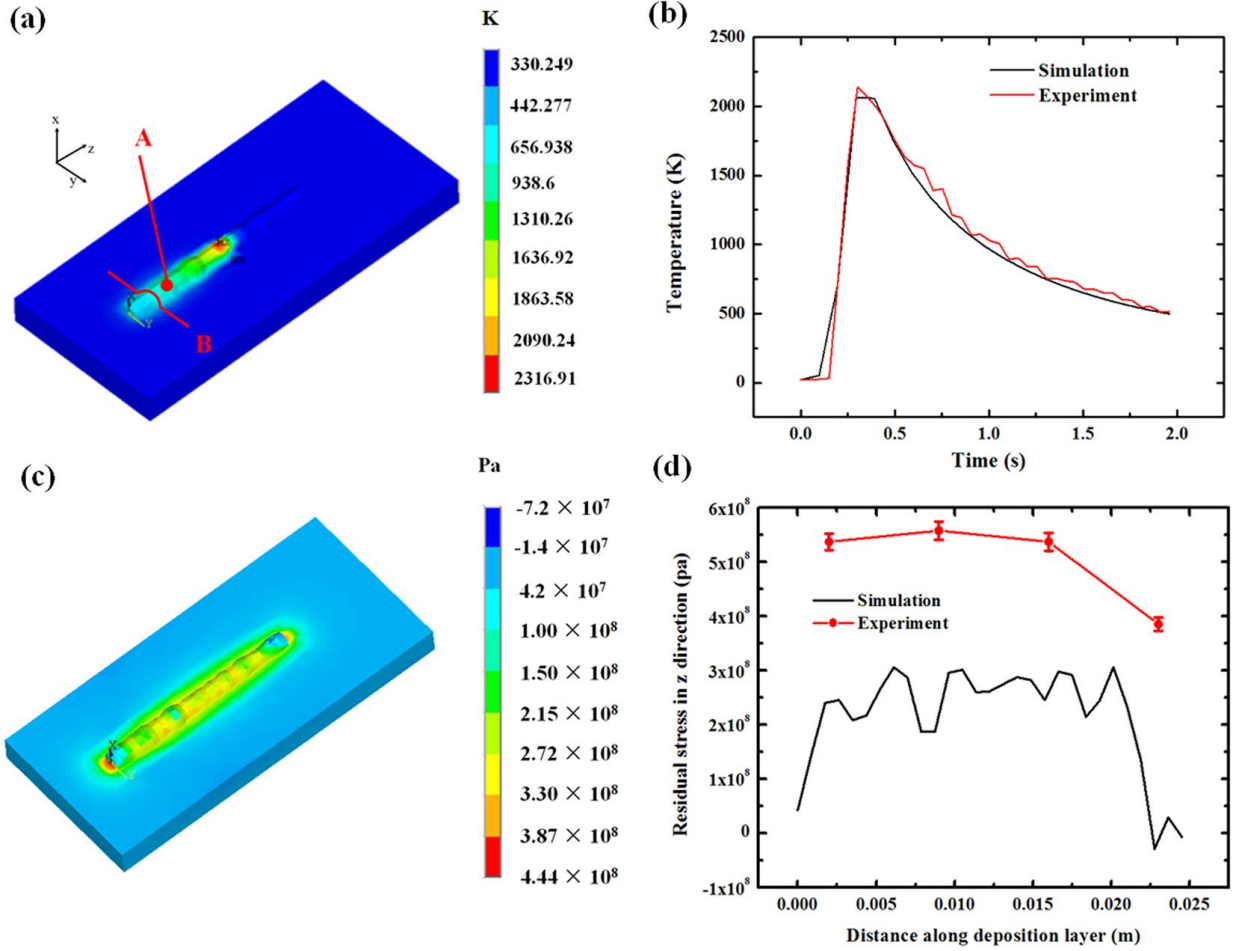
Temperature field simulated by thermal FEM method. (**a**) Temperature distribution at half of the process, (**b**) Time history of temperature at point A, (**c**) the z direction stress distribution, (**d**) The residual stress along the top surface of the sample.

It can also be observed from Fig. 5 that the height change at the beginning is more obvious than at other times. This result is because when material is first deposited on the cold substrate, it cools down very quickly, and the contraction is also more intense than that at later times. Figure 6 shows the temperature field of the melt pool and its surrounding areas. The high-temperature region is mainly concentrated in the deposited layer. The highest temperature in the melt pool area is approximately 2300 K, while the lowest part in the substrate is close to room temperature. Figure 6b shows the time history of temperature at point A. The simulation result matched well with the experimental results (measured by a two-colour pyrometer IMPAC ISR 12-LO/GS). It can be seen that when the laser approaches point A, the temperature rises steeply, and after it has passed, the temperature quickly drops down to 490 K in approximately 1.5 seconds. The high-temperature field and the high cooling ratio in the deposition layer indicate that the contraction mainly occurs in this part. To verify the FEM model, the final residual stress along the sample surface (Fig. 4a) has been measured through XRD method. As shown in the Fig. 6d, the trend of the experiment result and simulation results are similar. In both curves, the stress decreases at the end of the deposition layer.

**Figure 7:**
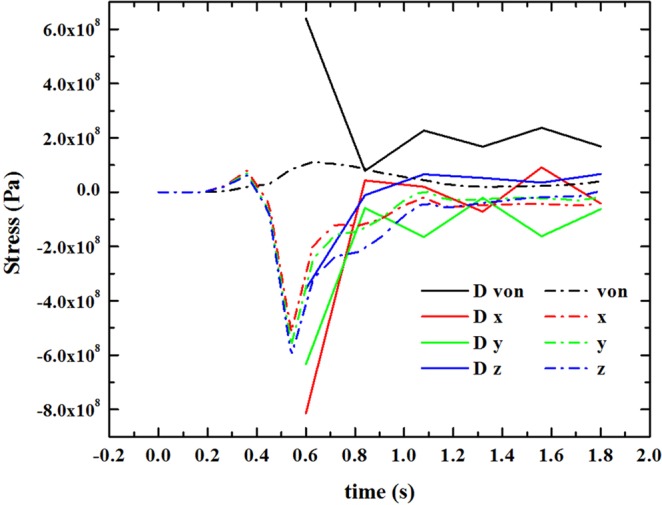
Time history of stress at point A.

Figure 7 compares the time history of stress at point A between the displacement method and thermal simulation. The results of the two methods are similar, and all the x, y and z components of the stress show similar trends throughout the deposition process. For the thermal simulation, the stress first remains at zero and then gently rises at 0.2 s. Just after 0.1 s, the curve drops down sharply, reaches the minimum point, and then quickly bounces up to a relatively stable value. For the displacement method, the stress value starts from the lowest value and then quickly soars up and remains stable, as the thermal simulation shows. The von Mises stress shows a similar trend in both methods. Although the stress in the displacement method appears suddenly at a very high value, the trends after the data appear are same: both curves drop down to their maximum value and reach a stable state.

When the molten pool approaches the observation point, the red, high-temperature molten pool first forms the material at the observation region. Then, some tensile stress is generated in the formed material due to the thermal expansion (corresponding to the stress increase around 0.38 seconds in Fig. 7). As the molten pool moves on, the expansion of the materials in front of the observation point compresses the materials behind it, creating a compressive stress at the observation point. When the heat source is far away, the materials cool down and contract intensively, which leads to a rapid decrease in the compressive stress.

The displacement method simulates the process when the melt pool passes over the observation point. Each time a new melt pool and displacement are applied, the melt pool drags the materials behind it. Similar to tightening a fixed string, the contraction in the front creates a tensile force along the surface, dragging all the materials behind it towards the melt pool.

**Figure 8:**
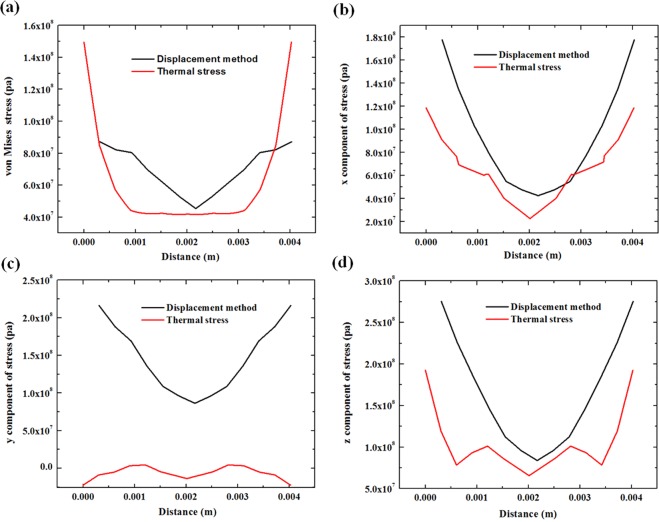
The stress distribution at the deposition/substrate interface in section B. (**a**) von Mises stress, (**b**) x component of stress, (**c**) y component of stress, (**d**) z component of stress.

Figure 8 displays the distribution of the von Mises stress and the x, y, and z components of the stress along the deposition/substrate interface at section B. In most conditions, the stress distribution is similar to a reverse parabola pattern in both methods, and the stresses calculated through the displacement method are slightly smaller than those obtained by the thermal method.

For the x and z components of stress, the stress value is much higher at the edge of the deposition layer, especially in the results of the thermal simulation method. The stress reaches the minimum value at the middle part of the section line. By contrast, the stress change is gentler in the displacement method.

In the y stress component, the distribution in the thermal method is close to other two directions. However, the distribution in the displacement method is different compared to other results. It has the lowest value of stress on the edge. From edge to centre, the stress first rises up to 1/3 the length of the section and then drops down to a local extreme in the middle of the section.

Since the high-temperature area is concentrated in the deposition layer, when it cools down, the surface of the deposition/substrate interface has the highest cooling ratio and contracts more intensely than do other parts. This phenomenon reflects the stress distribution on the deposition edge; both methods have a high stress concentration on the interface. In our calculation, the difference in the y component of stress in two methods is from taking the height change at the highest points in every direction. Therefore, by considering the volume contraction during the cooling process and the movement of the surface, the y-direction has been overestimated. This result is reflected in the different stress distribution in the y-direction. This problem can be solved when all of the surface displacements have been recorded and applied.

**Figure 9:**
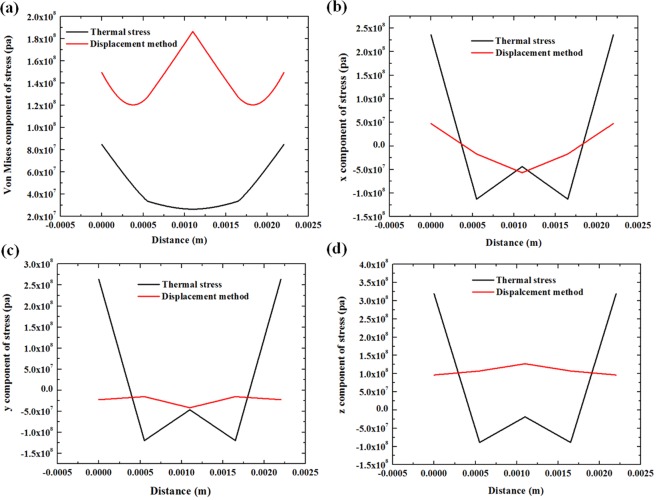
The stress distribution along the surface of the deposition layer in section B. (**a**) von Mises stress, (**b**) x component of stress, (**c**) y component of stress, (**d**) z component of stress.

Figure 9 compares the stress distribution along the surface of section B when the laser is at the end of the deposition layer. Although the trend of stress distribution on the surface is not very similar to that of the thermal method, at some critical points, the value is similar. At both the surface and bottom, the von Mises stresses are higher than those in the thermal method, and they can be used as safe criteria to judge whether dangerous stress is generated and may cause failure.

**Figure 10:**
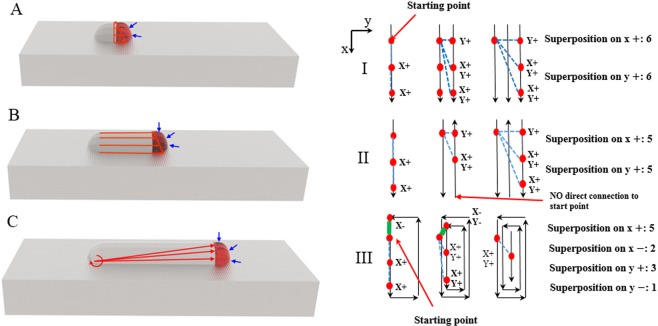
The stress superposition during the laser deposition.

Similar results obtained through two different methods reveal the fact that the volume change from the melt pool to solid state plays an important role in the evolution of stress. Therefore, we can deduce that the change in stress during the laser deposition is like the process shown in Fig. 10. In step A, each time the melt pool moves, the contraction of the molten pool drags the dry/wet interface and generates a set of gradient forces. In step B, as the deposition layer forms, the force is spread over the deposited material, which generates tensile stress inside the material. In step C, as the height and length of the deposited layer grows, torque is generated at the far end of the layer. Thus, a stress superposition process occurs during the laser deposition. Accordingly, we can roughly judge whether a scan strategy is suited for a certain product. For example, as shown in Fig. 10 for 3 different scan routes, we can estimate the stress superposition at the start point. We take a hypnosis that the shrinkage of the melt pool only occurs 3 times along the long edge and that each time the melt pool shrinks, it drags the materials around it. Obviously, the superposition at the start point in route II is 1 time less in both the X+ and Y+ directions compared to that of route I. In route III, there is 3 times shrinkage along the long edge, 2 times shrinkage on the inside long edge and 1 time shrinkage in the final scan. Therefore, for route III, the superposition in the X+ direction is 5 times and in the Y+ direction is 3 times; it also has 2 times the stress superposition in X− direction and 1 times the stress superposition in the Y− direction, which will release some stress in the X+ and Y+ direction. Therefore, taking all factors into consideration, route III has the least stress concentration, route II ranks second and route I has the highest stress concentration at the start point.

The camera calibration, image processing, height measurement, and FEM model building routines are run with MATLAB software, and it is used to submit the job to the FEM software to do the final calculation. The program is run on a laptop with 16 GB RAM and a i7-3615QM 2 core CPU. The single-step wall time is 2 seconds. With the current equipment, the computation time cannot compete for the manufacturing speed. However, the system can still do 2 second delay monitoring by dividing the deposited layer into 2 sections and performing a 2-step calculation. There is still a lot of space to compress the calculation time, such as using a more efficient programming language. Additionally, the calculation speed can be doubled or tripled by applying a more powerful computer and further increased with the development of computer hardware.

## Conclusions

The main goal of this article is to investigate a new method to monitor the stress change during the laser deposition process. This method uses a CCD camera to measure the height of the melt pool and a line laser to measure the height of the solidified parts. By using the change of surface displacement during the solidification, the stress inside the deposition layer is calculated. The results can be summarized in the following points.

1. The height of the melt pool and its solid state have been successfully measured through computer vision. The surface displacement mainly occurs during the solidification process. After solidification, there is no obvious size change in the solidified layer during the cooling process.
2. From the melt pool to the solid state, the shape of the deposition layer does not change dramatically. The surface curvatures of the two states are similar to each other; the height of the solidification layer is slightly lower than that of the melt pool.
3. The results of the time history of stress and the stress distribution in displacement method and thermal simulation are similar. The deformation of liquid-solid and solid-solid happens less than 0.0166 s, just after the laser passed.
4. Each time the melt pool contracts, it drags the materials around it, causing stress to accumulate throughout the deposited material. The idea of superposition of stress during the laser deposition can be used to do an approximate optimisation of the scan route. By calculating the time of stress superposition at some critical point, one can quickly decide the best strategy for scanning.

## Parameter of X-ray diffraction (XRD)

The parameters of XRD stress test were listed in Table 1.

**Table 1:** Parameter of XRD stress test.

**Target**: Mn (Kα avg 2.1031 Angstroms)	**Oscillation**(s): Beta 4.00°
**Target Power**: 18 kV, 4 mA	**Collection Time**: 3 second × 10 exposures
**Gain Material**: β Titanium Shim	**Peak Fit**: Gaussian 85%
**Gain Power**: 11.5 kV, 4 mA	**Two Peak Model**: No
**X-Ray Elastic Constant**: 20,199 ksi	**LPA Correction On**: Yes
**Crystallographic Plane**: {211}	**LPA Correction On**: Yes
**Bragg Angle (2θ)**: 152.8°	**Gain Correction**: P/G

## Supplementary information

Dataset 1
