# Supplementary material for: Online Stress Measurement During Laser-aided Metallic Additive Manufacturing
Source: Sci Rep. 2019 May 21;9:7630. doi: 10.1038/s41598-019-39849-0 (PMC6529430; doi:10.1038/s41598-019-39849-0)
Supplement: Supplementary file 1 — Dataset 1 [file 41598_2019_39849_MOESM1_ESM.pdf]

Online Stress Measurement During Laser-aided Metallic Additive Manufacturing

Yi Lu[1], Guifang Sun[1], Xianfeng Xiao[3] & Jyoti Mazumder[2]

Supplementary data.

Another two sets of experiments have been done to verify the displacement method.

The applied laser power is 1500 W, the scan speed is 0.5 Inch/s and laser diameter is 1 mm. The protection gas is nitrogen and the gas flow rate is 40 CFH. As shown in Fig I c, the length of the sample is 10.7 mm. Fig. I a shown the height of the two sample. Fig. I b is the residual stress along the sample surface. Fig. II a-c is the stress distribution at the deposition/substrate interface at cross-section c in fig. I c . The trend of the stress distribution is similar to the thermal simulation.

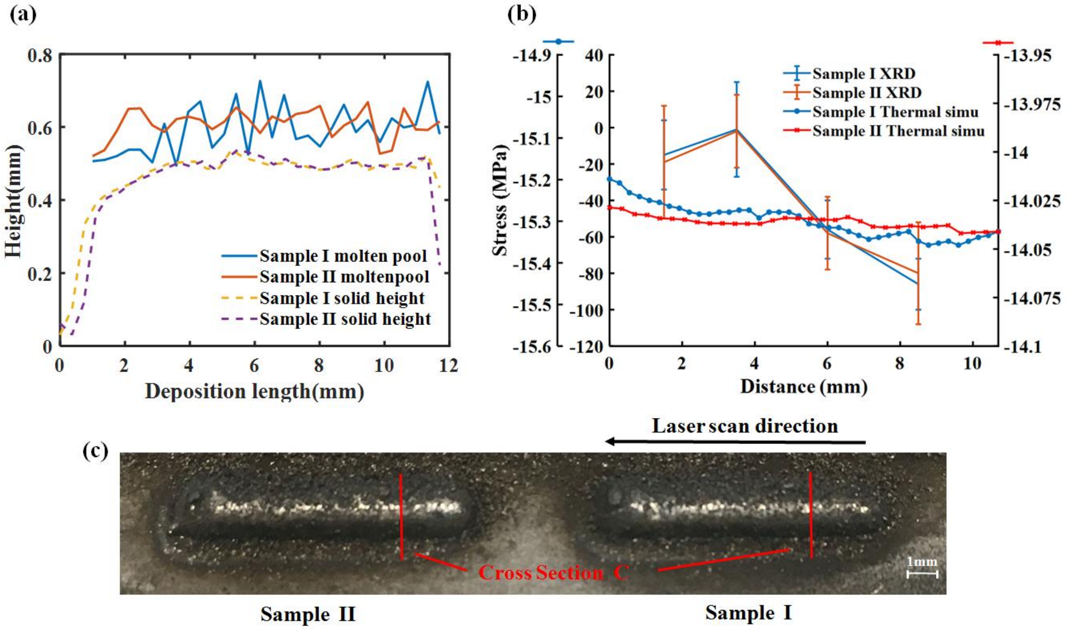

Fig. I (a) Molten pool height and solidified height, (b) Final residual stress along the sample surface.

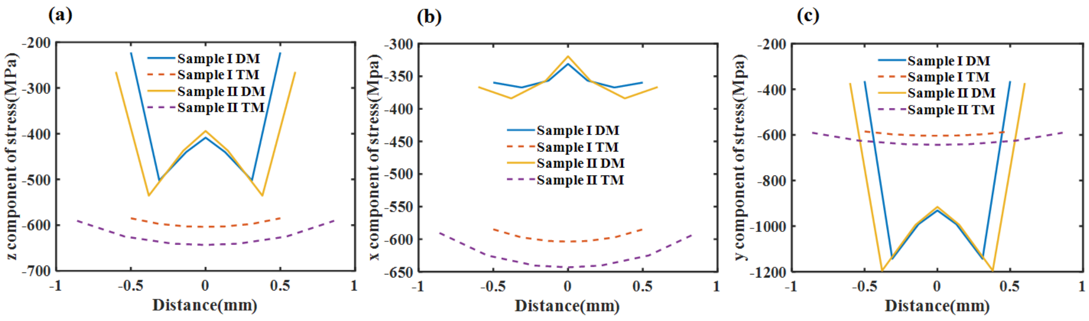

Fig. II The stress distribution at the deposition/substrate interface in section C, calculated by Displacement method(DM) and Thermal simulation(TM). (a) z component of stress, (b) x component of stress, (c) y component of stress.

1 College of Mechanical Engineering, Southeast University Nan Jing, 210000, China. 2 Department of Mechanical Engineering, College of Engineering, University of Michigan, Ann Arbor, 48109-2136, USA. 3 College of Mechanical and Vehicle Engineering, Hunan University Chang Sha, 410082, China. Correspondence and requests for materials should be addressed to Jyoti Mazumder (email: mazumder@umich.edu)
